# A Simple Cell-Based Assay Reveals That Diverse Neuropsychiatric Risk Genes Converge on Primary Cilia

**DOI:** 10.1371/journal.pone.0046647

**Published:** 2012-10-03

**Authors:** Aaron Marley, Mark von Zastrow

**Affiliations:** Departments of Psychiatry and Cellular and Molecular Pharmacology, University of California San Francisco, San Francisco, California, United States of America; Institut Curie, France

## Abstract

Human genetic studies are beginning to identify a large number of genes linked to neuropsychiatric disorders. It is increasingly evident that different genes contribute to risk for similar syndromes and, conversely, the same genes or even the same alleles cross over traditional diagnostic categories. A current challenge is to understand the cellular biology of identified risk genes. However, most genes associated with complex neuropsychiatric phenotypes are not related through a known biochemical pathway, and many have an entirely unknown cellular function. One possibility is that diverse disease-linked genes converge at a higher-level cellular structure. The synapse is already known to be one such convergence, and emerging evidence suggests the primary cilium as another. Because many genes associated with neuropsychiatric illness are expressed also outside the nervous system, as are cilia, we tested the hypothesis that such genes affect conserved features of the primary cilium. Using RNA interference to test 41 broadly expressed candidate genes associated with schizophrenia, bipolar affective disorder, autism spectrum disorder and intellectual disability, we found 20 candidates that reduce ciliation in NIH3T3 cells when knocked down, and three whose manipulation increases cilia length. Three of the candidate genes were previously implicated in cilia formation and, altogether, approximately half of the candidates tested produced a ciliary phenotype. Our results support the hypothesis that primary cilia indeed represent a conserved cellular structure at which the effects of diverse neuropsychiatric risk genes converge. More broadly, they suggest a relatively simple cell-based approach that may be useful for exploring the complex biological underpinnings of neuropsychiatric disease.

## Introduction

Human disease genetics are presently making important inroads to investigating the biological basis of severe neuropsychiatric disorders. As progress in this exciting field continues to advance, a major next challenge is to understand biological mechanisms underlying the influence of identified genes on pathophysiology or disease vulnerability. This requires defining normal cellular structures or functions that are impacted by the respective disease-linked genes.

A remarkable observation that has emerged is that the same locus, the same gene or even the same allele can affect risk for phenotypically different syndromes [1], [2], [3], [4], [5], [6]. Many genes implicated in schizophrenia (SCZ), for example, are also implicated in bipolar affective disorder (BAD) [7], [8], [9], [10], [11]. The same can be said for genes implicated in autism spectrum disorder (ASD) and intellectual disability (ID) [12]. Such extensive genetic crossover suggests that common biological vulnerabilities underlie the pathophysiology of, or determine susceptibility to, multiple neuropsychiatric syndromes. Identifying these shared ‘nodes’ of vulnerability is not a simple task because the genetic architecture of neuropsychiatric illness is complex. Indeed, most genes exhibiting such phenotypic crossover do not encode products that are known to physically interact or function in a shared biochemical pathway. This suggests that apparently disparate risk genes may be related at a higher level of biological integration, through convergent effects on a common cellular structure or process.

One such shared cellular node, which is already well recognized, is the synapse. Numerous genes associated with severe neuropsychiatric disorders affect synaptic structure or function [13], [14]. Even though their respective gene products are not related by direct interaction, or through any single biochemical pathway, these genes converge in supporting the integrated operation of synapses in intercellular signaling. Only a subset of neuropsychiatric risk genes are known to affect synapses, however. Moreover, synapses are specific to neural cell types, whereas neuropsychiatric disorders have systemic manifestations and many disease-implicated genes are expressed also outside the nervous system [15]. This raises the question of whether there exist additional cellular nodes at which disease-linked genes converge, and if such convergence of disease-linked genes can be observed in non-neural cells.

Here we describe a cell culture-based approach for addressing these questions, and provide evidence suggesting that the primary cilium represents such a node. Specifically, we identify 20 genes previously linked to diverse neuropsychiatric disorders -including SCZ, BAD, ASD and ID - which converge in supporting proper cilium formation or maintenance in a simplified non-neural model system.

## Results

We showed previously that knocking down expression of DISC1, a gene linked to SCZ, BAD and ASD, disrupts primary cilia in medium spiny neurons cultured from rat striatum. We also established that DISC1 is endogenously expressed in NIH3T3 cells, an experimentally advantageous non-neural cell model, and that the ciliation defect of DISC1 knockdown could be reliably observed in this cell type [16]. Accordingly, in the present study we extended our search to ask if primary cilia are affected by knockdown of other broadly expressed genes linked to major neuropsychiatric disorders, sampling a variety of candidates and verifying their endogenous expression in NIH3T3 cells. First, we considered disease-linked genes encoding proteins already known to physically interact with DISC1 [17], [18], [19], [20]. Second, we sampled genes whose protein products are not known to interact with DISC1, and whose disease associations have been identified through rare variants distinct from DISC1. Third, we investigated a sampling of genes whose disease associations have been detected independently in large genome-wide association studies (**Tables 1** **,** **2** **, and** **3** **, respectively**).

**Table 1:** Summary of DISC1 interactors tested.

Gene symbol	SCZ	ASD	BAD	ID	Cilia phenotype	Full name	Proposed function
CEP63				21983783		centrosomal protein 63 kDa	centrosome, cytoskeleton
CEP170	21926974					centrosomal protein 170 kDa	centrosome, cytoskeleton
FEZ1	1552225, 21926974					fasciculation and elongation protein zeta 1	cytoskeleton
PDE4B	16293762					phosphodiesterase 4B	cAMP hydrolysis
SYNE1		20351715	21572417, 21738484 21926972, 22565781		19596800	spectrin repeat containing, nuclear envelope 1	cytoskeleton

**Table 2:** Summary of rare variants tested.

Gene symbol	SCZ	ASD	BAD	ID	Cilia phenotype	Full name	Proposed function
ABCA13	19944402	22495311	19944402			ATP-binding cassette, sub-family A (ABC1), member 13	transporter
ASPM				12355089, 15355437, 16673149, 14722158 19028728, 9770472 20978018		asp (abnormal spindle) homolog, microcephaly associated (Drosophila)	Regulation of mitotic spindle
CCDC18		22495311				coiled-coil domain containing 18	unknown
CHD1		22495311				chromodomain helicase DNA binding protein 1Probable transcription regulator	Transcription regulator
CHD3		22495309				chromodomain helicase DNA binding protein 3	Transcription regulator
CHD5	See 1p36	See 1p36 One gene 22495309	See 1p36	See 1p36		chromodomain helicase DNA binding protein 5Probable transcription regulator	Transcription regulator
CHD7		22495309, 1563772 1635959, 15688419		21378379		chromodomain helicase DNA binding protein 7	Transcription regulator
CNTN4		19404257, 18349135 18551756				contactin 4	Cell surface interaction
CNTNAP2	17646849	16571880, 19896110				contactin associated protein-like 2	unknown
FOXP1		20950788,21572417 22521361		20950788, 20848658		forkhead box P1	Transcription regulator
GNB1L	See 22q11	See 22q11, Also 2 genes, 16684884	See 22q11	See 22q11, Also 1 gene, 22095694		guanine nucleotide binding protein (G protein), beta polypeptide 1-like	G protein beta subunit
KATNAL2		22495311, 22495309				katanin p60 subunit A-like 2	Microtubule severing
KIF17	20646681					kinesin family member 17	Microtubule motor
NRXN1	18668038, 17989066 19197363,18945720 19736351	20468056,19736351 18179900, 20531469 17322880, 20162629		20468056, 19736351 19896112		neurexin 1	Cell surface interaction
TBX1	See 22q11	See 22q11, Also 2 genes 1668488		See 22q11		T-box 1	Transcription regulator
22q11 Deletion Syndrome	7644464, 18668038 19557195, 18668039 10509171	16684884, 19557195 20020400, 18252227 7455106, 20531469 19046189, 17322880		22095694, 16208694, 20020400			
1p36 Deletion Syndrome	19805367	8914743, 19015223 20034100, 16840569 1075546, 15689456		9326330, 18245432, 12687501, 16835933, 20034097			

**Table 3:** Summary of common variants tested.

Common Variants	SCZ	ASD	BAD	ID	Cilia phenotype	Full name	Proposed function
ANK3	2018514, 21926974	22521361	18711365, 20351715 21926972, 21926974			ankyrin 3, node of Ranvier (ankyrin G)	cytoskeleton
CACNA1C	21926974	15454078	18711365, 21926972 21926974			calcium channel, voltage-dependent, L type, alpha 1C subunit	Calcium channel
CCDC68	21926974, 21791550					coiled-coil domain containing 68	unknown
CNNM2	21926974					Ancient conserved domain-containing protein 2	Divalent metal cation transporter
CSMD1	21926974					CUB and Sushi multiple domains 1	Unknown, type I membrane protein
HISTH2BJ	19571808, 19571809					histone cluster 1, H2bj	Chromatin structure
IFT88			20713499		11062270	intraflagellar transport 88 homolog	Component of intraflagellar transport complex
MIR137	21926974					microRNA 137	Gene regulation
MMP16	21926974					matrix metallopeptidase 16 (membrane-inserted)	Endopeptidase for ECM substrates
NEK4	21926972 21926974		21926972, 21926974 19416921		21685204	Serine/threonine-protein kinase Nek4	kinase
NRGN	19571808 21791550					neurogranin	Signaling mediator
NOTCH4	1,957,180,819,571,810					notch 4	Signaling receptor
NT5C2	21926974					5′-nucleotidase, cytosolic II	Hydrolysis of purine nucleotides
ODZ4			21926972			odz, odd Oz/ten-m homolog 4 (Drosophila)	Signal transduction
PGBD1	1,957,180,819,571,810					piggyBac transposable element derived 1	unknown
PRSS16	1957180, 9571809					protease, serine, 16 (thymus)	protease
SDCCAG8	21926974				20835237	serologically defined colon cancer antigen 8	Polarity, ciliogenesis
STT3A	21926974					STT3, subunit of the oligosaccharyltransferase complex, homolog A (S. cerevisiae)	N-glycosylation
TCF4	19571811, 19571808 21926974	:22521361	17478476	20713499		transcription factor 4	Transcription factor, wnt signalling
TRANK1			22182935, 21926972			tetratricopeptide repeat and ankyrin repeat containing 1	unknown
TRIM26	21926974					tripartite motif containing 26	unknown

Numbers indicate Pubmed ID for primary references to disease associations or cilia phenotype, as indicated. Gene name and proposed function are excerpted from the Uniprot database.

**Figure 1:**
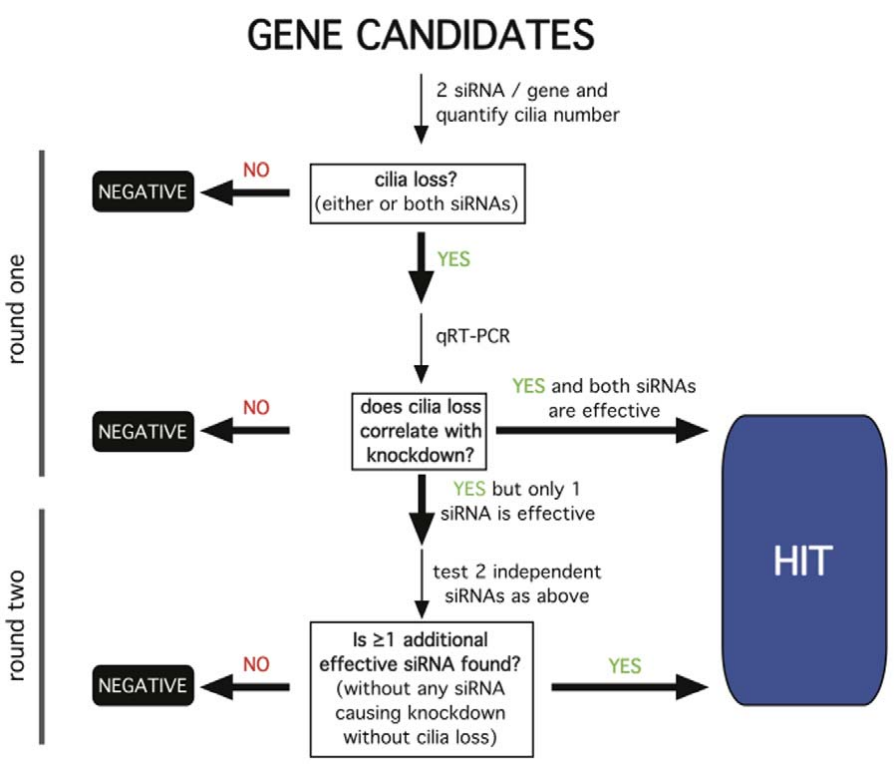
Schematic of the siRNA screening strategy. The work flow was organized into two rounds of screening. If both duplexes produced cilia loss and knocked down gene expression as assessed by qRT-PCR, the gene was scored as a hit. If only one duplex was effective, and only this duplex knocked down expression, a second round was pursued as indicated. All other outcomes were scored as a negative. A similar strategy was pursued for increased ciliation and, for genes whose manipulation produced this phenotype, additional analysis of ciliary length was conducted as described in the text.

Candidate genes were knocked down in NIH3T3 cells by specific siRNA transfection. Knockdown was assessed using quantitative reverse transcription- polymerase chain reaction (qRT-PCR). Fluorescence microscopy was used to assess the effects of gene knockdown on the fraction of cells in the culture expressing a primary cilium. We designed a screening strategy (**Fig. 1**) that required any gene scored as a hit to meet three critical experimental criteria: First, the knockdown effect on ciliation must be reproducible using at least two independent RNA duplexes. Second, endogenous expression of the gene must be verified in the cell model. Third, the observed effects of each RNA duplex on ciliation must correlate with the degree of knockdown achieved.

**Figure 2:**
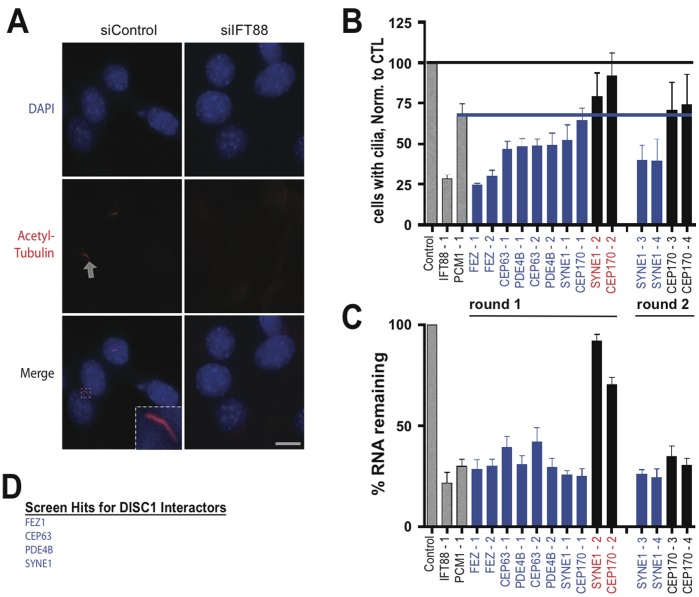
Screening candidate genes encoding DISC1 interactors. (**A**) Representative example fluorescence micrographs showing the analysis of ciliation. NIH3T3 cells were fixed 72 hours after RNA transfection, nuclei were identified by DAPI stain (top row, shown in blue) and primary cilia were detected by acetylated tubulin immunoreactivity (middle row, shown in red). Arrow indicates an example. Lower row shows merged images. The inset shows the example of a typical cilium, as indicated by arrow, at higher magnification. Scale bar, 10 um. (**B**) Compiled results from the screen of candidate genes. Normalized percent ciliation was determined for cells transfected with each RNA duplex and compiled across multiple experiments (n = 5) and specimens (>150 cells/condition/experiment), as described in *Materials and Methods*. First three bars from the left (coded in gray) indicate negative (scrambled duplex) and positive controls (IFT88 and PCM-1 knockdown). The degree of cilia depletion produced by PCM-1 depletion was chosen as the threshold for scoring a positive ciliation defect for the unknowns (blue line). (**C**) Relative transcript level observed in siRNA-transfected cells was determined by qRT-PCR analysis and normalized to transcript level in cells transfected with control (scrambled) duplex. Each RNA duplex is indicated in the abscissa, and the mean and standard deviation from triplicate determinations of transcript level are plotted on the ordinate as ‘% RNA remaining’. Blue bars indicate duplexes that met criteria both for reduced ciliation and produced gene knockdown. Duplexes indicated in red text represent ‘splits’ that were carried into a second round for screening with two additional duplexes. SYNE1 passed the second round and CEP170 did not. (**D**) Summary list of genes scored positive for ciliation defect.

Acetylated tubulin immunoreactivity was used as a cilia marker, and DAPI was used to stain nuclei (see Experimental Procedures). In untransfected cultures, or cultures transfected with control (scrambled, nonsilencing) duplex, 50–60% of cells recognized by DAPI stain (**Fig. 2** **A**, top row) possessed a clearly visible primary cilium recognized by acetylated tubulin immunoreactivity (**Fig. 2** **A**, middle row). The arrow in the figure indicates an example, which is shown at higher magnification in the merged image (**Fig. 2** **A**, bottom row). As expected [21], a previously validated [16] siRNA targeting IFT88 caused a visually obvious decrease in the number of ciliated cells (compare left and right panels). To quantify effects on ciliation, the fraction of ciliated cells observed in siRNA-transfected cultures was determined relative to that observed in control (scrambled RNA) -transfected cultures in the same experiment, and this normalized value was then averaged across multiple experiments. Quantification across multiple specimens verified that IFT88 knockdown reliably produced a pronounced (∼75%) reduction in the number of cells expressing a primary cilia (**Fig. 2B**, compare first and second bars from the left). Knockdown of PCM1, another positive control [22], [23], also reliably reduced ciliation albeit to to a lesser degree (∼35%; **Fig. 2B**, compare first and third bars from the left). We chose this degree of ciliary depletion, which exceeded four standard deviations from the effect any negative control RNA duplex, as a conservative threshold for scoring positive hits with siRNAs targeting the candidate genes (**Fig. 2** **B**, blue line).

The majority of candidate genes that encode DISC1-interacting proteins strongly inhibited ciliation when knocked down. FEZ1, CEP63 and PDE4B were identified in round one, based on the criterion of both duplexes producing a ciliation defect that correlated with knockdown verified by qRT-PCR (**Fig. 2** **B** and **C**). SYNE1 was called in round two due to a non-silencing duplex in round one (Syne1-2, **Fig. 2C**), followed by confirmed effects of independent duplexes in round two. A split was also observed in round one for CEP170 but, in contrast to SYNE1, the independent duplexes tested in round two failed to reduce % ciliation below our experimental threshold; accordingly CEP170 was not scored as a hit. Thus, using this conservative assay strategy, four of the five disease-associated DISC1 interactors tested produced a clearly detectable loss of ciliation when knocked down (**Fig. 2** **D**): CEP63, FEZ1, PDE4B and SYNE1.

**Figure 3:**
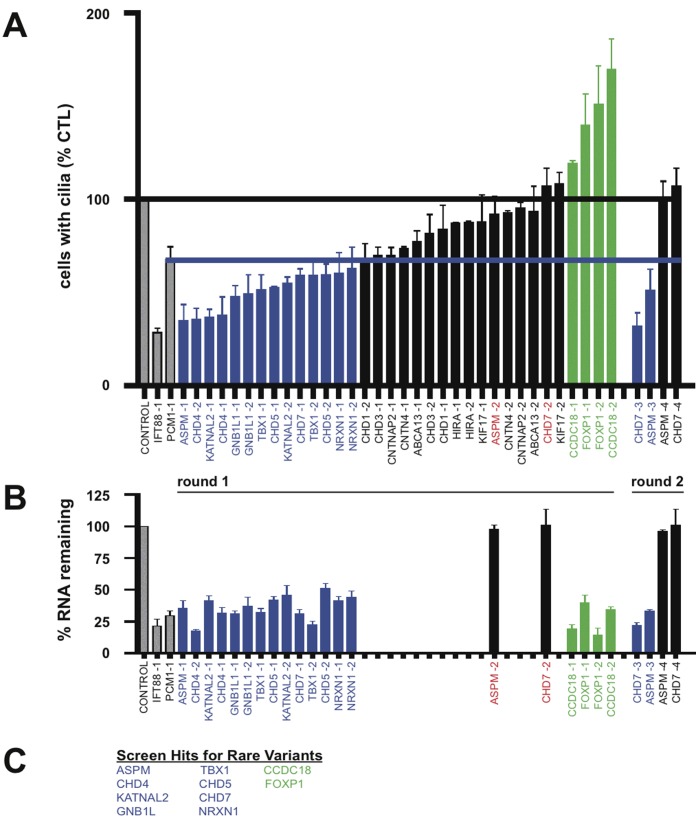
Screening rare variants. (**A**) Compiled ciliation results for the candidates listed in the abscissa, carried out and indicated in the figure as described in Fig. 2 B. (**B**) Endogenous expression and candidate gene knockdown assessed by qRT-PCR analysis. All of the siRNA duplexes that produced a ciliation defect were tested for knockdown (blue bars). Green bars indicate duplexes that increased % ciliation, and these were also tested by qRT-PCR. Correlative qRT-PCR analysis was also carried out on all ‘splits’ to determine if the duplex not producing a ciliation defect also failed to knock down expression. Those splits for which this was true (black bars with duplex identity indicated in red) were carried into the second round (the same strategy as described in Fig. 2). (**C**) Summary list of hits characterized by decreased % ciliation indicated in blue, and hits characterized by increased % ciliation indicated in green.

A number of genes whose protein products are not known to interact with DISC1, and whose disease linkage was detected through rare variants distinct from DISC1, also affected ciliation when knocked down. In our survey we tested 18 and, using the same experimental strategy and scoring criteria as for the direct DISC1 interactors, identified seven more disease-linked genes that reduced ciliation when depleted (**Fig. 3** **A** and **B**). Six of these were called in round one and two more in round two (**Fig. 3** **C**): ASPM, CHD4, CHD5, CHD7, GNB1L, KATNAL2, NRXN1 and TBX1.

**Figure 4:**
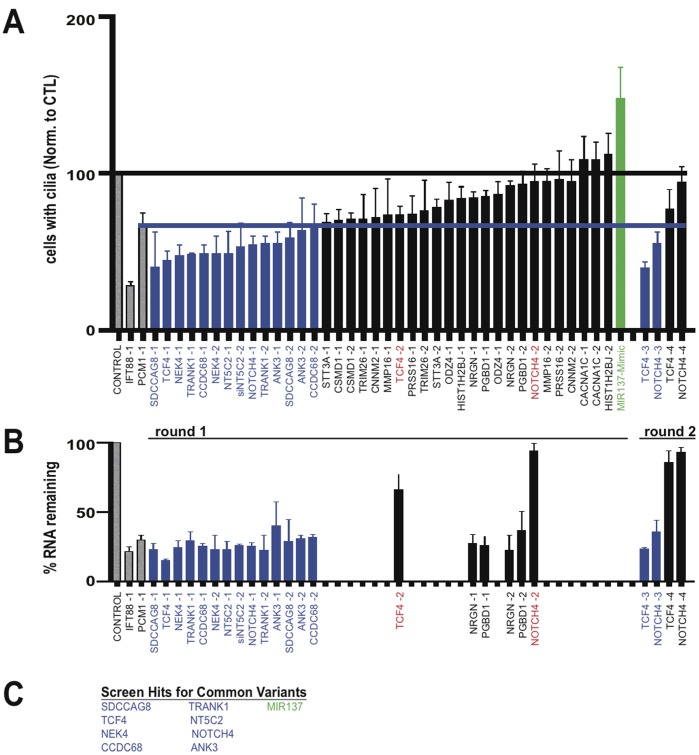
Screening common variants. (**A**) Compiled ciliation results for the candidates listed in the abscissa, carried out and indicated in the figure as described in Fig. 2 B. (**B**) Endogenous expression and candidate gene knockdown assessed by qRT-PCR analysis. The color coding scheme for scoring of duplexes is the same as in previous figures. All duplexes supporting a hit were tested by qRT-PCR and a subset of duplexes targeting genes scored as negatives were spot-checked as indicated. (**C**) Summary list of hits characterized by decreased % ciliation indicated in blue, and hits characterized by increased % ciliation indicated in green.

We identified still more hits by sampling broadly expressed genes that have been independently implicated in neuropsychiatric disease through large GWAS efforts. Specifically, of the 19 genes tested in our limited screening effort, eight strongly reduced ciliation when knocked down (**Fig. 4** **A** and **B**): ANK3, CCDC68, NEK4, NOTCH4, NT5C2, SDCCAG8, TCF4 and TRANK1 (**Fig. 4C**).

**Figure 5:**
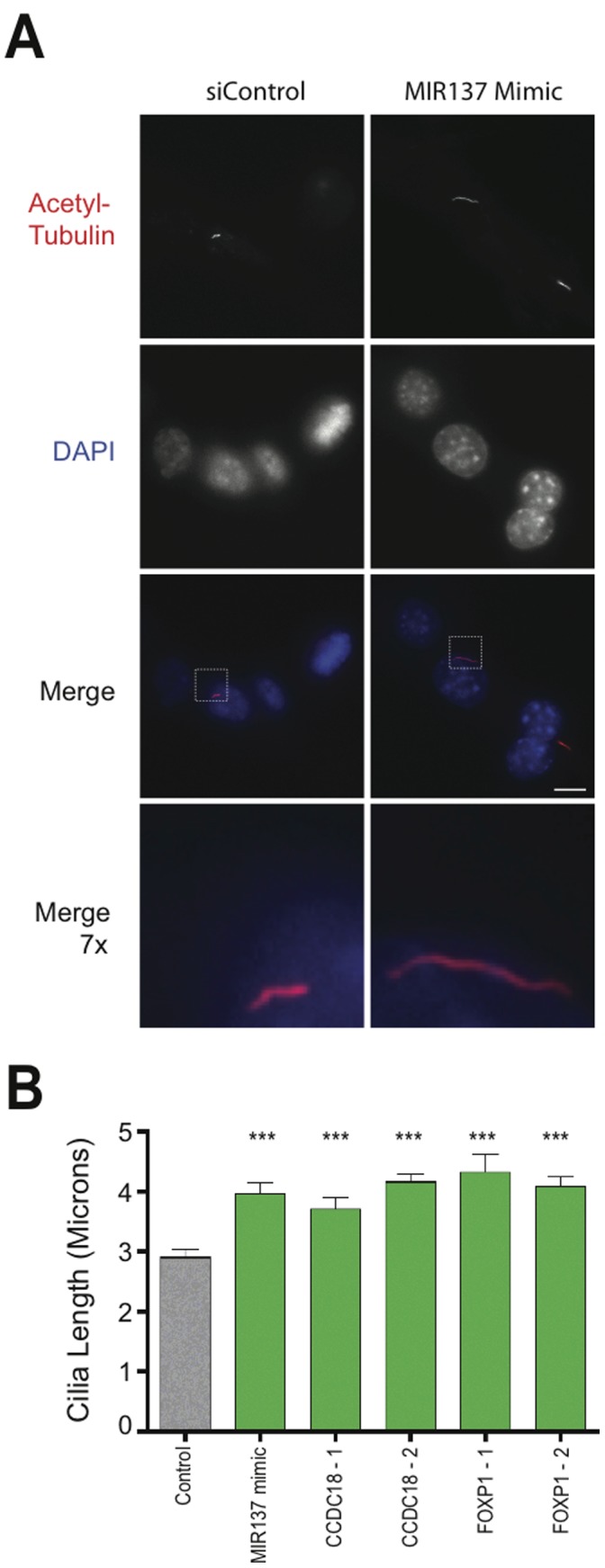
Hits that increase % ciliation also increase cilia length in NIH3T3 cells. (**A**) Example of ciliary lengthening produced by MIR137 mimic. Cells were transfected with scrambled siRNA control (‘siControl’, left column) or MIR137 mimic (right column). Example images of acetylated tubulin immunoreactivity (top row) and DAPI fluorescence (second row from top) are shown. Merged images are displayed in the third row and, the indicated areas at 7× higher magnification in the fourth row. Scale bar, 10 µm. (**B**) Cilia length was measured in randomly selected fields as described in *Materials and Methods*. Average cilium length for the indicated experimental conditions is indicated by the bar, and error bar indicate S.E.M. (scrambled siRNA Control, N = 45; MIR137 Mimic, N = 43; siCCDC18-1, N = 31; siCCDC18-2, N = 46; siFOXP1-1, N = 38; siFOXP1-2, N = 27). ***, P<0.0001 by two-tailed unpaired Student’s t-test.

In carrying out the screen, we also noticed that some siRNAs had an opposite effect- they appeared to increase the frequency of ciliation observed in transfected cultures. To verify this, we applied the same criteria as used in the primary screen for cilia loss, except we required at least two independent duplexes to produce a significant increase in % ciliation, and we excluded any candidate for which this phenotype did not correlate with knockdown assessed by qRT-PCR. The genes whose knockdown produced this effect were CCDC18 and FOXP1 (green bars in **Fig. 3A and B**). We also noticed increased % ciliation in cells transfected with the MIR137 mimic (green bars in **Fig. 4A and B**). This effect was visually striking in some of the transfected cells (**Fig. 5A** shows an example for the MIR137 mimic) and a significant, albeit moderate, increase in average cilia length (relative to the scrambled control) was verified for all of the hits in this group (**Fig. 5B**).

## Discussion

In the present study we identified 20 genes linked to phenotypically diverse clinical syndromes including SCZ, BAD, ASD and ID, which disrupt primary cilia when knocked down in a simple cell culture model, and three for which altering cellular expression increases the frequency of ciliation and cilia length. Three of the genes identified- NEK4, SDCCAG8 and SYNE1- were previously reported to function in ciliogeneisis [24], [25], [26], supporting the validity of the present screening approach and revealing a ciliary phenotype for 20 additional candidates. This is a remarkably high hit rate relative to that observed in RNAi screens not focusing on neuropsychiatric risk genes, and we note that screening the druggable genome collection for ciliary defects achieved <1% hit rate using similar methods and scoring criteria [27]. With the exception of DISC interactors and CHD4/CHD5 [28], we are not aware of previous evidence for physical or functional interaction between the hits identified in the present study. Accordingly, we propose that the present cell-based screen has the potential to detect additional relationship(s) between disease genes, and that primary cilia may represent a common cellular ‘node’ at which the effects of diverse neuropsychiatric risk genes converge.

We pursued a simple loss-of-function approach, based on RNAi-mediated knockdown in a non-neural cellular model, and thus focused on detecting conserved normal cellular functions of the selected candidate genes. We did not attempt to test specific disease-linked alleles, or disease-relevant neuronal populations, although doing so could represent an interesting future direction. Accordingly, the present results do not indicate that neuropsychiatric disorders represent ciliopathies per se. Indeed this seems unlikely, as major neuropsychiatric syndromes are not associated with classical manifestations of gross cilia loss or dysfunction. Rather, the present results suggest that primary cilia represent a common cellular node at which diverse disease-linked genes impact proper formation, maintenance or regulation. Thus we would propose that neuropsychiatric pathology could involve relatively subtle defects in the structure or function of primary cilia, rather than a complete disruption, much like disease-linked genes affecting the synapse do not simply abrogate neurotransmission in affected individuals.

The primary cilium represents a complex and dynamic cellular structure that is conserved in neurons and non-neural cell types, and which mediates diverse and important functions in cellular signaling [29]. Primary cilia display numerous receptors and downstream signaling components that are important both in development and adult tissue function, including D1 and D2 dopamine receptors which are affected by drugs used currently in the management of neuropsychiatric illness [16], [30]. Moreover, while classical ciliopathies and primary neuropsychiatric disorders are discrete clinical entities, many ciliopathies have behavioral manifestations [31]. Thus we think that ciliary convergence of diverse neuropsychiatric risk genes, as suggested by the present analysis, is plausible with regard to previous knowledge in the area.

The present study took a simplified cell culture-based approach that was motivated by 1) extensive conservation of primary cilia across cell types, 2) the fact that many of genes implicated in neuropsychiatric disease are expressed widely in neural as well as non-neural tissues, and 3) previous evidence showing that NIH3T3 cells reliably detect DISC1-dependent ciliation effects. A possible implication of our results is that primary cilia contribute directly to neuropsychiatric pathophysiology and/or determine disease vulnerability. Alternatively, because primary cilia are themselves complex structures that are functionally associated with numerous cellular processes, our results could reflect a more distant convergence of disease-linked genes [32], [33]. In either case, our results establish a starting point for elucidating the cellular function of genes determining neuropsychiatric disease risk. They may also provide a useful approach for identifying biochemical events occurring downstream of disease-linked gene convergence, and thus reveal new targets for therapeutic consideration.

## Materials and Methods

### Cell Culture and Transfection

NIH3T3 cells (ATCC, Manassas, VA) were maintained in Dulbecco’s modified Eagle’s medium supplemented with 10% fetal calf serum (University of California, San Francisco, Cell Culture Facility). The fraction of ciliated cells declined with extended passaging, so all experiments were carried out using early-passage cells within 30 days of thaw.

### RNA Depletion

Predesigned siRNA duplexes were ordered from (Qiagen). A complete list of siRNA target sequences is provided in Table S1. Duplexes were transfected using Lipofectamine RNAi-max (Invitrogen) using the optimized protocol provided by the manufacturer for NIH3T3 cells. In all experiments reagent amounts were scaled to 12 well plates, based on the optimized protocol listed for 24-well plates. Experiments were conducted 3 days after siRNA transfection followed 6 hours of starvation. For each gene selected for RNA analysis cell lysates from NIH3T3 cells were run in triplicate through Qiashredder columns (Qiagen) and RNA was purified and concentrated in RNeasy MinElute columns (Qiagen). Using Brilliant II SYBR Green dye chemistry (Qiagen), qRT-PCR was performed with the Stratagene MX4000. Data from the assay were standardized and normalized with MxPro software (Stratagene). A complete list of amplification primers is presented in Table S2.

### Fluorescence Microscopy

Cells were fixed with 3.7% formaldehyde dissolved in PBS and permeabilized with 0.1% Triton X- 100 and 3% milk in PBS. We incubated cells with mouse anti-acetylated tubulin (Sigma, 1 µg/ml for 60 min) and then probed with goat anti-rabbit Alexa594 (Invitrogen) for 20 minutes. For cilia counts in NIH3T3 cells, we stained for acetylated tubulin in NIH3T3 cells indicated above. Across experiments, under control (scrambled RNA duplex) conditions, 55±5% cells formed primary cilia. This was determined by counting the number of primary cilia labeled with acetylated tubulin and dividing by the number of DAPI-positive cells examined. To assure unbiased analysis, in all cases microscopic fields with similar cell density were first selected at random by DAPI staining, and then acetylated tubulin immunoreactivity was examined to score cilia. Each siRNA condition was measured in the same manner. For each duplex, experiments were preformed at least 5 times on separate days. In each experiment, ≥150 cells were examined for each condition. Specimens were imaged by epifluorescence microscopy using a Nikon inverted microscope, 60×, numerical aperture 1.4 objective (Nikon), mercury arc lamp illumination and standard dichroic filter sets (Chroma). Images were captured using a cooled CCD camera (Princeton Instruments) and exposures adjusted to avoid saturation. For display, acquired images were converted to 8 bit format with ImageJ software (http://imagej.nih.gov/ij/) using linear lookup tables. Cilia length was measured in fluorescence images as described [34].

### Statistical Analysis

Quantitative data were averaged across multiple independent experiments, with the number of experiments specified in the corresponding figure legend. Unless indicated otherwise, the error bars represent the S.E.M. calculated across experiments calculated using Prism 4.0 software (GraphPad Software, Inc.).

## Supporting Information

Table S1 **Compiled siRNA target sequences.**
(XLS)

Table S2 **Compiled qRT-PCR primer sequences.**
(XLS)
